# Supplementary material for: Prevalence of traditional uvulectomy and its associated factors among parents who had less than 6 months of infants in Gondar City, north-west Ethiopia: Mixed study design
Source: PLoS One. 2024 Jul 5;19(7):e0305175. doi: 10.1371/journal.pone.0305175 (PMC11226079; doi:10.1371/journal.pone.0305175)
Supplement: Attachment — Submitted filename: PONE-D-23-22864_reviewer-1.pdf [file pone.0305175.s002.pdf]

PLOS ONE

# Prevalence of traditional uvulectomy and its associated factors among parents who had less than 6 months of infants in Gondar city, north-west Ethiopia: Mixed study design

--Manuscript Draft--

| | |
|---|---|
| **Manuscript Number:** | PONE-D-23-22864 |
| **Article Type:** | Research Article |
| **Full Title:** | Prevalence of traditional uvulectomy and its associated factors among parents who had less than 6 months of infants in Gondar city, north-west Ethiopia: Mixed study design |
| **Short Title:** | Prevalence of traditional uvulectomy |
| **Corresponding Author:** | Lakachew Yismaw Bazezew, MSc<br>Debre Tabor University<br>Debre Tabor, Amhara regional state ETHIOPIA |
| **Keywords:** | Keywords: Prevalence, traditional uvulectomy, associated factors, infants, Gondar city, Ethiopia |
| **Abstract:** | Abstract<br>Background: Traditional uvulectomy is widely practiced in Africa especially in sub-Saharan countries including Ethiopia. Limited Studies conducted in different times and areas of the world have shown that the prevalence of uvulectomy and its associated factors are varied from country to country. There is limited evidence to determine the prevalence and reasons of mal practice in Ethiopia. Therefore, this study aimed to assess the prevalence of traditional uvulectomy, and its associated factors among parents who had children aged less than 6 months.<br>Method s: a community-based cross-sectional mixed study design was conducted among 634 participants selected by a systematic sampling technique. The collected data were entered into Epi-data, and analyzed using STATA version 14software. Descriptive statistics were computed and presented using tables, figures and texts. Factors associated with the prevalence of uvulectomy were selected for multiple logistic regressions at the probability value (p value) of less than 0.2 in the X2-analysis. Statistically significant associated factors were identified at probability value (p value) less than 0.05 and adjusted OR (AOR) with a 95% CI. A total of 10 interviewees were included using purposive sampling techniques for qualitative study and thematic content analysis was employed to analyze the data.<br>Results: The prevalence of traditional uvulectomy was 84.60% (CI: 81.77%n - 87.42%). Being rural residence (adjusted OR=2.08, CI=1.06 - 4.12), mothers aged 30 to 34 years (adjusted OR=2.9, CI=1.28 - 6.60), having no antenatal care visit (adjusted OR=3.91, CI= 1.33 - 11.47), having no bad obstetric history (adjusted OR=2.11, CI=1.12 - 3.98) , having no postnatal care visit (adjusted OR=3.88, CI=1.36 - 11.12) and mothers' poor attitude (adjusted OR=3.32, CI=2.01 - 5.47) were statistically significant associated factors of traditional uvulectomy. Seeking traditional uvulectomy, lack of information and third party pressure were the main themes of the qualitative study that support the findings of quantitative study.<br>Conclusion: the prevalence of traditional uvulectomy was high. Being rural residence, mothers aged 30 to 34 years, having no antenatal care visit, having no complications of obstetric history, having no postnatal care visit and mothers with poor attitude were statistically significant associated factors of traditional uvulectomy. Lack of information about uvulectomy and third-party pressure was the reasons for seeking traditional uvulectomy. |
| **Order of Authors:** | Lakachew Yismaw Bazezew, MSc |
| | Asrat Hailu Dagne, MSc(Asistant prof) |
| | Destaye Guadie Kassie, MSc(asistant prof) |
| | Abebaw Alemayehu Desta, MSc(Asistant prof) |
| | Mengistu Berhan Gobeza, MSc(Asistant prof) |

Powered by Editorial Manager® and ProduXion Manager® from Aries Systems Corporation

| Additional Information: | |
|---|---|
| **Question** | **Response** |
| **Financial Disclosure**<br><br>Enter a financial disclosure statement that describes the sources of funding for the work included in this submission. Review the submission guidelines for detailed requirements. View published research articles from *PLOS ONE* for specific examples.<br><br>This statement is required for submission and **will appear in the published article** if the submission is accepted. Please make sure it is accurate.<br><br>**Unfunded studies**<br>Enter: *The author(s) received no specific funding for this work.*<br><br>**Funded studies**<br>Enter a statement with the following details:<br>• Initials of the authors who received each award<br>• Grant numbers awarded to each author<br>• The full name of each funder<br>• URL of each funder website<br>• Did the sponsors or funders play any role in the study design, data collection and analysis, decision to publish, or preparation of the manuscript?<br>• **NO** - Include this sentence at the end of your statement: *The funders had no role in study design, data collection and analysis, decision to publish, or preparation of the manuscript.*<br>• **YES** - Specify the role(s) played.<br><br>* typeset | The author(s) received no specific funding for this work. |
| **Competing Interests**<br><br>Use the instructions below to enter a competing interest statement for this submission. On behalf of all authors, disclose any competing interests that could be perceived to bias this work—acknowledging all financial support and any other relevant financial or non-financial competing interests. | The authors have declared that no competing interests exist. |

| | |
|---|---|
| This statement is required for submission and **will appear in the published article** if the submission is accepted. Please make sure it is accurate and that any funding sources listed in your Funding Information later in the submission form are also declared in your Financial Disclosure statement.<br><br>View published research articles from *PLOS ONE* for specific examples.<br><br>**NO authors have competing interests**<br><br>Enter: *The authors have declared that no competing interests exist.*<br><br>**Authors with competing interests**<br><br>Enter competing interest details beginning with this statement:<br><br>*I have read the journal's policy and the authors of this manuscript have the following competing interests: [insert competing interests here]*<br><br>* typeset | |
| **Ethics Statement**<br><br>Enter an ethics statement for this submission. This statement is required if the study involved:<br><br>• Human participants<br>• Human specimens or tissue<br>• Vertebrate animals or cephalopods<br>• Vertebrate embryos or tissues<br>• Field research<br><br>Write "N/A" if the submission does not require an ethics statement.<br><br>General guidance is provided below. Consult the submission guidelines for detailed instructions. **Make sure that all information entered here is included in the** | Ethical approval was obtained from the Ethical Committee of University of Gondar, College of Medicine and Health Sciences. A formal letter of cooperation was written for Gondar City health offices and kebeles, and permission to conduct the study was obtained from the offices and the Kebeles. Participants were informed that they had the right to withdraw from the study at any time. Moreover, we informed the purpose, procedures, advantages and disadvantages. Finally, informed written consent was obtained from each study participant. |

Powered by Editorial Manager® and ProduXion Manager® from Aries Systems Corporation

| | |
|---|---|
| **Methods section of the manuscript.**<br><br>**Format for specific study types**<br><br>**Human Subject Research (involving human participants and/or tissue)**<br>• Give the name of the institutional review board or ethics committee that approved the study<br>• Include the approval number and/or a statement indicating approval of this research<br>• Indicate the form of consent obtained (written/oral) or the reason that consent was not obtained (e.g. the data were analyzed anonymously)<br><br>**Animal Research (involving vertebrate animals, embryos or tissues)**<br>• Provide the name of the Institutional Animal Care and Use Committee (IACUC) or other relevant ethics board that reviewed the study protocol, and indicate whether they approved this research or granted a formal waiver of ethical approval<br>• Include an approval number if one was obtained<br>• If the study involved *non-human primates*, add *additional details* about animal welfare and steps taken to ameliorate suffering<br>• If anesthesia, euthanasia, or any kind of animal sacrifice is part of the study, include briefly which substances and/or methods were applied<br><br>**Field Research**<br><br>Include the following details if this study involves the collection of plant, animal, or other materials from a natural setting:<br>• Field permit number<br>• Name of the institution or relevant body that granted permission | |
| **Data Availability**<br><br>Authors are required to make all data underlying the findings described fully available, without restriction, and from the time of publication. PLOS allows rare exceptions to address legal and ethical concerns. See the PLOS Data Policy and FAQ for detailed information. | No - some restrictions will apply |

Powered by Editorial Manager® and ProduXion Manager® from Aries Systems Corporation

| | |
|---|---|
| A Data Availability Statement describing where the data can be found is required at submission. Your answers to this question constitute the Data Availability Statement and **will be published in the article**, if accepted.<br><br>**Important:** Stating 'data available on request from the author' is not sufficient. If your data are only available upon request, select 'No' for the first question and explain your exceptional situation in the text box.<br><br>Do the authors confirm that all data underlying the findings described in their manuscript are fully available without restriction? | |
| **Describe where the data may be found in full sentences. If you are copying our sample text, replace any instances of XXX with the appropriate details.**<br><br>• If the data are **held or will be held in a public repository**, include URLs, accession numbers or DOIs. If this information will only be available after acceptance, indicate this by ticking the box below. For example: *All XXX files are available from the XXX database (accession number(s) XXX, XXX.*).<br>• If the data are all contained **within the manuscript and/or Supporting Information files**, enter the following: *All relevant data are within the manuscript and its Supporting Information files.*<br>• If neither of these applies but you are able to provide **details of access elsewhere**, with or without limitations, please do so. For example:<br><br>*Data cannot be shared publicly because of [XXX]. Data are available from the XXX Institutional Data Access / Ethics Committee (contact via XXX) for researchers who meet the criteria for access to confidential data.*<br><br>*The data underlying the results* | The dataset used and/or analyzed during the current study are available from the corresponding author on reasonable request |

Powered by Editorial Manager® and ProduXion Manager® from Aries Systems Corporation

| | |
|---|---|
| *presented in the study are available from (include the name of the third party and contact information or URL).*<br>• This text is appropriate if the data are owned by a third party and authors do not have permission to share the data.<br><br>* typeset | |
| Additional data availability information: | |

Manuscript Click here to access/download;Manuscript;Manuscript .docx

# Prevalence of traditional uvulectomy and its associated factors among parents who had less than 6 months of infants in Gondar city, north-west Ethiopia: Mixed study design

Lakachew Yismaw Bazezew [1*], Asrat Hailu Dagne[2], Destaye Guadie Kassie[3], Abebaw Alemayehu Desta[4], Mengistu Berhan Gobeza[3]

[1]Department of pediatric and neonatal nursing, Debre Tabor University, Debre Tabor, Amhara Region, Ethiopia

[2]Department of Midwifery, Debre Tabor University, Debre Tabor, Amhara Region, Ethiopia

[3]Department of pediatrics and child health, School of Nursing, University of Gondar, Gondar, Amhara Region, Ethiopia

[4]Department of Surgical Nursing, School of Nursing, University of Gondar, Gondar, Amhara Region, Ethiopia

Corresponding author: Lakachew Yismaw Bazezew

**Email address**

Lakachew Yismaw Bazezew:lakachewyismaw@gmail.com Institutional email:Lakayism@dtu.edu.et

Asrat Hailu Dagne: 1221asrat@gmail.com Institutional email: asrahail@dtu.edu.et

Destaye Guadie:dstgd32@gmail.com

Abebaw Alemayehu:abexsosy@gmail.com

Mengistu Berhanu:mbirhanu11@gmail.com

**Abstract**

**Background:** Traditional uvulectomy is widely practiced in Africa especially in sub-Saharan countries including Ethiopia. Limited Studies conducted in different times and areas of the world have shown that the prevalence of uvulectomy and its associated factors are varied from country to country. There is limited evidence to determine the prevalence and reasons of mal practice in Ethiopia. Therefore, this study aimed to assess the prevalence of traditional uvulectomy, and its associated factors among parents who had children aged less than 6 months.

**Methods:** a community-based cross-sectional mixed study design was conducted among 634 participants selected by a systematic sampling technique. The collected data were entered into Epi-data, and analyzed using STATA version 14software. Descriptive statistics were computed and presented using tables, figures and texts. Factors associated with the prevalence of uvulectomy were selected for multiple logistic regressions at the probability value (p value) of less than 0.2 in the $X^2$- analysis. Statistically significant associated factors were identified at probability value (p value) less than 0.05 and adjusted OR (AOR) with a 95% CI. A total of 10 interviewees were included using purposive sampling techniques for qualitative study and thematic content analysis was employed to analyze the data.

**Results**: The prevalence of traditional uvulectomy was 84.60% (CI: 81.77%n - 87.42%). Being rural residence (adjusted OR=2.08, CI=1.06 - 4.12), mothers aged 30 to 34 years (adjusted OR=2.9, CI=1.28 - 6.60), having no antenatal care visit (adjusted OR=3.91, CI= 1.33 - 11.47), having no bad obstetric history (adjusted OR=2.11, CI=1.12 - 3.98) , having no postnatal care visit (adjusted OR=3.88, CI=1.36 - 11.12) and mothers' poor

attitude (adjusted OR=3.32, CI=2.01 - 5.47) were statistically significant associated factors of traditional uvulectomy. Seeking traditional uvulectomy, lack of information and third party pressure were the main themes of the qualitative study that support the findings of quantitative study.

**Conclusion**: the prevalence of traditional uvulectomy was high. Being rural residence, mothers aged 30 to 34 years, having no antenatal care visit, having no complications of obstetric history, having no postnatal care visit and mothers with poor attitude were statistically significant associated factors of traditional uvulectomy. Lack of information about uvulectomy and third-party pressure was the reasons for seeking traditional uvulectomy.

**Keywords:** Prevalence, traditional uvulectomy, associated factors, infants, Gondar city, Ethiopia

## Background

Traditional uvulectomy is the total or partial removal of the uvula by the traditional healer and it is cutting of the uvula and sometimes the nearby structures like, tonsils (1-4). A small soft tissue that hangs down from the back of the mouth above the throat of the two tonsils is the uvula. Uvula influences the tone of voice, it moisten the throat or pharyngeal mucosa, it secret large amount of thin serous saliva which lubricates Oro-pharyngeal mucosa in conjunction with the soft palate, it closes the nose-pharynx, and it prevents aspiration/regurgitation of food or water through the nose which helps to prevent choking during swallowing, it boosts immunological function, and it is used in producing sounds necessary for communication (5-7).

Traditional instruments like sharp blades and threads are used for the cutting of the uvula (8-10). The tools used to cut the uvula are not sterile and the procedure conducted in carelessly by traditional ways, which expose to a variety of health problems that could lead to local or systemic complications like speech problems, injury to the tongue, broken teeth, deformity of teeth, excessive bleeding lead to anemia and respiratory distress, long term dental problems, sepsis, tetanus, HIV/AIDSs, swallowing difficulty, malnutrition, otitis media, septicemia, and hepatitis (6, 11).

The prevalence of traditional uvulectomy was varies in developing countries and it was varies from 2.6% to 90% in Nigeria, Niger, and Ethiopia (10, 12-21). The prevalence of infant mortality and morbidity are caused by different factors including immediate and chronic complications of traditional uvulectomy (22-24).

Different studies indicate that parents' and traditional practitioner's attitude, family income, being rural residence, having had no antenatal care, antenatal and postnatal

couple counseling about traditional neonatal uvulectomy, home delivery, being house wife, maternal educational status, occupation, being rural residence, social outlook, parental perception towards uvulectomy, lack of effective modern treatment and third party pressure are among the factors that contribute to traditional uvulectomy practice (1, 12, 25, 26).

Despite efforts to reduce the problem over years, gaps still exist. Findings from studies in different regions of Ethiopia showed that the prevalence still have high and many of the study participants reported that the victims are vulnerable to various health problems. Traditional uvulectomy can cause serious complications and the reason is cultural, which can lead to infant death, especially in sub-Saharan countries including Ethiopia (19, 26) .

The recommendations to explore the factor related to traditional uvulectomy, evidences related to traditional uvulectomy are limited. As a result, identifying the prevalence of traditional uvulectomy in infancy and its associated factors have public health importance. This study will help as additional source of evidence after exploring local factors and prevalence of the problem. It will have contribution in improving the health of infants, changing parents' health behavior, and positive impacts on health institutions.

The traditional uvulectomy practice is cultural and varies from local to local or place to place and predictor variable like previous bad obstetric history were not considered so far. Moreover, the findings of this study will help program managers, stakeholders and health service providers to design appropriate interventions to prevent traditional uvulectomy. It will also be helpful to develop interventions aimed at reducing infant and child morbidity and mortality through increasing community awareness of uvulectomy as

harmful traditional practice. Therefore, this study aimed to assess the prevalence of traditional uvulectomy and its associated factors among parents of children aged less than 6 months in Gondar city, north-west Ethiopia 2022.

## Methods

### Study design, period and setting

A community-based concurrent mixed study design was conducted from October 4 to November 4, 2022. The study was conducted in Gondar city, which is located 182 kilometer away from the regional city Bahir Dar, and 748 kilometer from the capital city Addis Ababa. Gondar is one of the ancient and densely populated cities in Ethiopia and is located at an altitude of 12.4 North and 27.21 East. According to the recent administration, the city has 6 administration areas which consist of 36 kebeles. The city has 25 urban and 11 rural administrative kebeles. According to the 2023 Gondar city administrative estimation plan of vital registration, the total population was 475,172; among these around 251, 466 and 223, 706 were females and males, respectively. The estimated numbers that were mothers in the city who had infants less than 6 months of age were 3136. The city has eight health centers, 50 private clinics, one private hospital, one public comprehensive specialized referral hospital, and one primary public hospital.

### Participants

Parents of infants less than 6 months who were living at least six month in Gondar city were considered for quantitative study. Participants who were ill and unable to respond during the study period were excluded from the study. All mothers who had infants aged less than 6 months in Gondar Town were the study population. The sample size was calculated using single population proportion formula. The required sample size for this study was determined using the following assumptions; desired precision (d) = 5% , Confidence level = 95% ($Z\alpha/2$ = 1.96 value) and 52.5% of the prevalence of traditional

uvulectomy in South Gondar Zone (12). Hence, the calculated sample size by considering 10% non- response rate was 634. The participants were selected using systematic sampling technique. Ten interviewees and five key informants were included for qualitative part of the study. Health extension workers and traditional healers were participated in the study as key informants.

**Data collection tool and procedures**

Sociodemographic and obstetric-related data collection tools comprised of structured questionnaires that were prepared after a thorough literature review for quantitative study. The local situation of the study area and the purpose of the study were considered for preparing the survey. Questionnaires were developed first in English then translated to Amharic which is the colloquial language of the respondents by language expert for ease of understanding of the respondents.

Senior nurses were engaged in data collection and they had previous experience in data collection. Training had been given to 4 data collectors and 2 supervisors for 3 days duration. It was focused on the questionnaire content to ensure consistency of data, obtaining consent, maintaining neutrality, privacy issues, personal relation and ethics in research. The pretest was carried out before the actual data collection period among 32 (5%) of parents who had infants aged less than 6 months in Maksegnit Town. Based on the result of the pretest, corrections of confusion were made before the actual data collection time and data were collected via face-to-interview. The filled questionnaires were checked daily for completeness and consistency of the responses to eliminate possible errors.

For qualitative part of the study, the in-depth interview guide was prepared first in English then translated to Amharic and retranslated back to English for consistency. One data collector (research assistance) who had the educational status of master with previous qualitative data collection experience was selected. He was trained to be familiar with the objective and the methodology of the research. The interviews were audio-recorded and the interview duration was between 45 minutes and 60 minutes. The participants' emotions and non-verbal communication were taken as field notes. Saturation was determined when there were multiple overlapping responses across participants.

**Operational definition**

**Traditional Uvulectomy:** removal of the uvula by traditional healer in the traditional way, this was measured from the report of parents and was coded 1 if parents reported that their infant had subjected to traditional uvulectomy; otherwise, it was coded 0 (12, 13).

**Knowledge:** knowledge related issues of the respondent was assessed by five yes/no knowledge related questions which was measured separately from the report of caregivers and coded 1 if the caregivers reported yes; otherwise, it was coded 0 (1, 27)

**Attitude (Good, Poor):** Those with greater than the mean score of 5 attitude related questions were coded as having a good attitude and the rest as having a poor attitude (1, 12, 27).

**Data processing and analysis**

The investigators and supervisors checked the data manually for completeness. Data were entered into EpiData version 3.1 then exported to STATA version 14 for statistical

analysis. Categorical variables were presented as frequency tables. Continuous variables were presented as descriptive measures, expressed as mean and standard deviation. A chi-square test was done to identify factors associated with the dependent variable. Independent variables with a probability value (P-value) of less than 0.2 in the Chi-square analysis were entered in the multivariable logistic regression model to identify the independent predictors of the traditional uvulectomy practice. Crude and adjusted odds ratios were used to identify the strength of the association at a 95% confidence interval. A P-value of less than 0.05 was used to decide the significance of the association.

Codes were created and described first for qualitative data. Categories were developed after defining the concepts. The number of categories was reduced by" collapsing those are similar or dissimilar into broader higher-order categories". Finally, the codes were ordered into different themes, and the main theme, themes, and subthemes

**Ethics approval and consent to participate**

Ethical approval was obtained from the Ethical Committee of University of Gondar, College of Medicine and Health Sciences. A formal letter of cooperation was written for Gondar City health offices and kebeles, and permission to conduct the study was obtained from the offices and the Kebeles. Participants were informed that they had the right to withdraw from the study at any time. Moreover, we informed the purpose, procedures, advantages and disadvantages. Finally, informed written consent was obtained from each study participant.

## Result

### Sociodemographic characteristics

During the study period, a total of 630 mothers or care takers who had infants less than 6 months were included in the study with a response rate of 99.36% and the non-response were due to incomplete information for some questionnaires. The mean age of mothers or caregivers and child were 29.68 years (SD ± 4.78) and 2.16 months (SD±1.07), respectively. The majority of the respondents, 465 (73.81%), and 555 (88.10%) were from urban and Orthodox religious followers, respectively. About 92% of the participants were married. The remaining frequencies and percentages of sociodemographic-related characteristics are presented on the next page (see Table 1).

**Table 1: Sociodemographic characteristics of parents of infants aged less than 6 months in Gondar City, Amhara Region, Ethiopia, 2022**

| Variables | Frequency (n=630) | Percent (%) |
|---|---|---|
| **Infants age** | | |
| 0-1 month | **423** | **67.1** |
| Greater than one month | 207 | 32.9 |
| **Maternal or care taker age** | | |
| 18-24 year | 80 | 12.7 |
| 25-29 year | 247 | 39.21 |
| 30-34 year | 188 | 29.84 |
| Higher than or equal to 35 year | 115 | 18.25 |
| **Mothers' educational status** | | |
| Unable to read and write | 43 | 6.83 |
| Able to read and write | 22 | 3.46 |
| $1^{st}$-$12^{th}$ grade | 359 | 56.98 |
| Diploma, degree and master | 206 | 32.7 |
| **Occupational status** | | |
| Farmer | 52 | 8.25 |
| House wife | 350 | 55.56 |
| Civil servant | 128 | 20.32 |
| Daily laborer | 33 | 5.24 |
| Merchant | 67 | 10.63 |
| **Marital status** | | |
| Single | 18 | 2.85 |
| Married | 584 | 92.7 |
| Divorced | 18 | 2.86 |
| Widowed | 10 | 1.59 |

**Obstetric related characteristics**

Of the total participants, 83.97% (529) had one or more prenatal care visits. However, about 71.08% (376) of them were not counseled about harmfulness of traditional infant uvulectomy. Regarding mothers' place of delivery, greater than 99% of participants gave birth at health facility. From all the participants, 84.6% (533) of mothers had at least one postnatal care visit, but 0nly 27.39% of them were counseled about harmfulness of traditional uvulectomy. Moreover, the other obstetric, information and attitude-related factors were reported on the table (see table 2).

**Table 2: Obstetric, information and attitude related characteristics of parents of infants aged less than 6 months in Gondar City, Amhara Region, Ethiopia, 2022**

| Variables | Frequency (n=493) | Percent (%) |
|---|---|---|
| **Parity of mother** | | |
| Primiparous | 209 | 33.17 |
| Multiparous | 421 | 66.83 |
| **Had ANC follow up** | | |
| Yes | 529 | 83.97 |
| No | 101 | 16.03 |
| **Had PNC follow up** | | |
| Yes | 533 | 84.6 |
| No | 97 | 15.4 |
| **Had bad obstetric history** | | |
| Yes | 68 | 10.8 |
| No | 562 | 89.2 |
| **Breast feeding** | | |
| Yes | 374 | 59.4 |
| No | 256 | 40.6 |
| **Attitude of participants towards** | | |
| Good | 211 | 33.5 |
| Poor | 419 | 66.5 |
| **Hospitable health professional** | | |
| Yes | 85 | 13.5 |
| No | 545 | 86.5 |
| **Heard harmfulness of uvulectomy** | | |
| Yes | 535 | 84.9 |
| No | 95 | 15.1 |

ANC, Antenatal Care; PNC, Postnatal Care

## Factors associated with traditional uvulectomy

According to the participants' response, 84.6% (95% CI: 81.77% -87.42%) of infants were victims of traditional uvulectomy. Place of residence, age of mothers, Religion, educational status, no antenatal care visit, no PNC visits, had bad obstetric history, heard harmfulness of uvulectomy, and mothers' attitude towards uvulectomy were the predictor variables found to be associated with traditional uvulectomy. These predictor variables were included in the multivariable model to identify the independent predictors of traditional uvulectomy.

The analysis revealed that participants being rural residence (adjusted OR=2.08, CI=1.06 - 4.12), mothers aged 30 to 34 years (adjusted OR=2.9, CI=1.28 - 6.60), having no antenatal care visit (adjusted OR=3.91, CI= 1.33 - 11.47), having no bad obstetric history (adjusted OR=2.11, CI=1.12 - 3.98) , having no postnatal care visit (adjusted OR=3.88, CI=1.36 - 11.12) and mothers' poor attitude (adjusted OR=3.32, CI=2.01 - 5.47) were statistically significant associated factors of postpartum hemorrhage (See table 3).

The analysis of data from interview generated three themes. The three themes were seeking traditional uvulectomy, lack of information and third party pressure were the main themes of the qualitative study that support the findings of quantitative study.

## Seeking traditional Uvulectomy

This main theme, which defined seeking traditional uvulectomy, is a means to seek treatment for infants when they are sick. This theme supports the quantitative part of the study finding that indicate participants' poor attitude towards uvulectomy exposed the participant for seeking traditional uvulectomy, thinking low cost of uvulectomy and set

aside harmfulness of traditional uvulectomy. Therefore, they seek traditional uvulectomy. This is described by one of the interviewees as follows:

*"We have information and know about traditional uvulectomy, even though traditional uvulectomy is better than the modern one. not only that, the cost for cutting ranges from 250 to 350 Ethiopian birr finally I recommend the government to help the traditional healer by gifting spatula, gloves, and new cutting materials to reduce the spread of infections (I-04)."*

**Lack of information**

One of health extension workers claimed that lack of information is one of the issues of traditional uvulectomy. She described that educating and training of the communality to boost knowledge on harmful traditional uvulectomy is crucial. This main theme supports the findings of quantitative study that disclose having no ANC and PNC are the factors increasing traditional uvulectomy due lack of getting health education on traditional uvulectomy during ANC and PNC visits. This is explained by one of the key informants:

"*As my opinion, the first prevention modalities are by educating and training the community. Second, if the event is proven as bad, legal action should be taken by the responsible body against individual who are not changed through education. In another way most people go to health institutes to treat their children, but infants may not be cured. Mothers want to be cut it due to the reason that if not mother's desired to continue the treatment. After three to four modern treatment options mothers were also going to cut it traditionally. If not cured by the modern one, they choose the traditional one (k-01).*"

**Third party pressure**

Third part pressure is defined as motivation of individual or community to participate in ceremony or events for the benefit of other parties. One of the interviewee described that the third party pressure leads to traditional uvulectomy

"*Today's modern treatment option cannot treat and prevent the problem after and before it occurs", "As I usually heard from my neighbors" when uvula elongated and swelled will lead child death. My family and neighbors made pressure on me and at that time, we seek to be cut the uvula (I-09)."*

**Table 3: Bivariate and multivariables analysis of associated factors of traditional uvulectomy among parents of infants aged less than 6 months in Gondar City, Amhara Region, Ethiopia, 2022**

| Variables | Uvulectomy | | Crude OR (95%CI) | Adjusted OR (95%CI) |
|---|---|---|---|---|
| | Yes | No | | |
| **Place of residence** | | | | |
| Urban | 382 | 83 | 1 | |
| Rural | 151 | 14 | ***2.34 (1.29-2.25) | ***2.08 (1.08-4.12)** |
| **Age of mothers** | | | | |
| 20-24 year | 62 | 18 | 1 | |
| 25-29 year | 203 | 43 | **1.37(0.74-2.56) | 1.66(0.79-3.49) |
| 30-34 year | 164 | 20 | ***2.43(1.21-4.91) | ****2.9(1.28-6.6)** |
| Higher than or equal to 35 year | 65 | 11 | **1.79(0.85-3.78) | 2.12(0.90-4.97) |
| **Had ANC follow up** | | | | |
| Yes | 436 | 93 | 1 | |
| No | 97 | 4 | ***5.17(1.85- | ****3.91(1.33-11.4)** |
| **Had bad obstetric history** | | | | |
| Yes | 46 | 22 | 1 | |
| No | 487 | 75 | **3.11(1.76-5.45) | ***2.11(1.12-3.98)** |
| **Had PNC follow up** | | | | |
| Yes | 441 | 92 | 1 | |
| No | 92 | 5 | ***3.83(1.52-9.71) | ****3.88(1.36-11.1)** |
| **Mothers' attitude toward uvulectomy** | | | | |
| Good | 150 | 61 | 1 | |
| Poor | 383 | 36 | ***4.33(2.75-6.81) | *****3.3(2.01-5.47)** |

*, P Value≤0.05; **, P Value≤0.01;**, P Value≤0.001

ANC, Antenatal Care; PNC, Postnatal Care

**Discussion**

The prevalence of traditional uvulectomy was determined and statistically significant associated factors of traditional uvulectomy were identified. The prevalence of traditional uvulectomy in the this study was higher than a study conducted in Axum, Ethiopia (13), Debre Birhan ,Ethiopia, 23.7% (25), and Nigeria (10, 17). This difference may be due to deference in the study periods which is greater than the expected gaps that is longer than 5 years between the conducted studies, and the study participants of the current study who were less than 6 month old infants rather than most other study groups who were under 5 which are less commonly exposed group for traditional uvulectomy.

The prevalence of traditional uvulectomy in this study was in line with a study conducted in plateau state Nigeria (28), and in East Shewa Zone at Fentale woreda, Ethiopia (29). The similarity may be due to study setting, and unimproved awareness creation, inadequate access and quality of health care service and it may also be due to the related situations of communities' attitudes and healthcare service. The prevalence of traditional uvulectomy for this study is lower than the prevalence of the study conducted in Jigawa state Nigeria. The possible reason for the difference may be due to deference in study periods which is longer than 5 years between the two studies. This might also be because of health-seeking behavior, lack of health facilities, quality health care and better development of infrastructure.

The odds of practicing traditional uvulectomy among participants of rural residence were 2.08 times more likely than participants of urban residence. A similar result was found in a study conducted in Ethiopia (15, 26). The possible reason of this may be the study

setting which causes to lower level of awareness and poor attitudes because of poor accesses to health care services and lack of access to social media towards traditional uvulectomy, on the contrary easily accessible to traditional healers.

This study revealed that the odds of traditional uvulectomy among participants aged 30 to 34 years were 2.9 times higher than participants aged 20-24 years.  This may be because most participants in this age group are more prone to adopt the experiences of traditional malpractices from the community and their poor attitude considering the advantage outweigh than its bad effects ,also outweigh previous good results  of malpractice than bad  effects of  it.

Mothers who had no ANC visit were almost 3.91 times more likely to have infants suffered from traditional uvulectomy than those who had ANC visit. This finding is similar to the study conducted in Nigeria and Ethiopia (1, 14, 26). This study result is also supported by the qualitative result of this study since the majority of the participants practiced traditional uvulectomy due to their unscientific perceptions and lack of awareness to the mal practice. This may be because of their less opportunity to be counseled towards traditional uvulectomy since there is not visit of health institution that may the reason to have lack awareness and poor attitudes to that mal practice.

The odds of traditional uvulectomy among mothers of infants who had no bad obstetric history were 2 times higher than mothers of infants who had bad obstetric history. This could be because of no repeated visit of health institution for the bad obstetric history that may not increase the awareness and attitude during ANC follow up to prevent uvulectomy. Since, they were less likely to visit health institution; it is true that there is less opportunity to be counseled towards traditional uvulectomy.

The odds of traditional uvulectomy practice among mothers who had no PNC visit were 3.88 times higher than mothers who had a PNC visit. The finding of this study is consistent with the study conducted in Ethiopia (1). This study result is also supported by the qualitative part of this study that revealed the majority of the participants practiced traditional uvulectomy due to their unscientific perceptions and lack of awareness of the malpractice. This is true that if there are no repeated visits to health institution, mothers may not increase their awareness and attitude level to prevent the experience of uvulectomy. In addition to this, it could be because of less opportunity to be counseled towards traditional uvulectomy.

Poor attitude of mothers of infants aged less than 6 months was statistically significant associated factors of traditional uvulectomy. This study was coincide with a study carried out in Ethiopia (15). This could be due to the mothers' perceptions towards traditional uvulectomy practice which pushes them to accept an effective alternative treatment modality as health care service. Thus the attitude of parents is crucial to decide which treatment option is best to treat their infant's illness.

**Limitation**

There could be the possibility of recall bias because mothers were asked to report some events of associated factors within months sometimes ago.

**Conclusion**

The prevalence of traditional uvulectomy was high. Being rural mothers, mothers aged 30 to 34 years, having no antenatal care visit, having no complications of obstetric history, having no postnatal care visit and mothers with poor attitude were statistically

significant associated factors of traditional uvulectomy. Lack of information about uvulectomy and third-party pressure was the reason for seeking traditional uvulectomy.


## Funding

The study was not funded by any funders

## Acknowledgment

We are thankful to the data collectors and all the study participants for their willingness to participate in the study. We would also like to thank the University of Gondar College of Medicine and Health Science School of Nursing for the facilitation of the program.

## References

1. Alebachew Bayih W MBB, Yeshambel Alemu A. The burden of traditional neonatal uvulectomy among admissions to neonatal intensive care units, North Central Ethiopia, 2019: a triangulated crossectional study. Plos one. 2020 July 9;15(7):e0234866.
2. Neuromuscular function of the soft palate and uvula in snoring and obstructive sleep apnea: a systematic review. American Journal of Otolaryngology. 2018 May 1;39(3):327-37.
3. Abdullahi M, Amutta SB. Traditional uvulectomy among the neonates: experience in a Nigerian tertiary health institution. Borno Med J(Online). 2016:16-20.
4. Kambale R M, Balibuno Y, Francisca NI, Kasengi JB, Mayele GF, Masumbuko BM. Traditional uvulectomy, a common practice in South Kivu in the Democratic Republic of Congo. Medecine et sante tropicales. 2018 Apr 1;28(2):176-81.
5. Adebola S O, Ogunkeyede SA, Obebe FA, Olaniyan OD, Fawole OB, Salman A. Profile of pediatric traditional uvulectomy in North-West Nigeria: the need for caution and education. International Journal of Pediatric Otorhinolaryngology. 2016;88:194-8.
6. Balibuno Y, Kasengi B, Mayele F. Traditional uvulectomy, a common practice in South Kivu in the Democratic Republic of Congo. Medecine et sante tropicales. 2018 May 1;28(2):176-81.
7. Lange S, Mfaume D. The folk illness kimeo and “traditional” uvulectomy: an ethnomedical study of care seeking for children with cough and weakness in Dar es Salaam. Journal of Ethnobiology and Ethnomedicine. 2022 Dec;18(1):1-4.

8. Abdullahi A A. Influence of Indigenous Post-Natal Practices on Lactating Mothers' Emotional and Psychological Stability in Municipal Local Government Area of Kano State, Nigeria (Doctoral dissertation, Kwara State University (Nigeria)). .
9. Vecchiato N L. Traditional medicine. InThe ecology of health and disease in Ethiopia. Routledge. 2019 Jul 11:157-78.
10. Olajide T G, Gabriel-Alayode OE, Olajuyin OA, Adeniyi AM, Adegbiji AW. Prevalence and Clinical Profile of Traditional Uvulectomy in Ekiti, South West, Nigeria. Am J Otolaryngol Head Neck Surg. 2020;2020:1099.
11. LK S K, Khalam SA, Kurien NM. Uvulectomy; Symptomatic relief for chronic irritating cough and obstrutive apnoea syndrome in long uvula. Journal of Case Reports in Practice. 2014;2(1):9-11.
12. Yirdaw B W, Gobeza MB, Tsegaye Gebreegziabher N. Practice and associated factors of traditional uvulectomy among caregivers having children less than 5 years old in South Gondar Zone, Amhara Region, Ethiopia, 2020. 2022 Dec 22;17(12):e0279362.
13. Gebrekirstos K, Fantahun A, Buruh G. Magnitude and reasons for harmful traditional practices among children less than 5 years of age in Axum Town, North Ethiopia, 2013. International Journal of pediatrics. 2014 Jun 19;2014.
14. Farouk Z L, Gambo S, Sani H, Kabir H, Ibrahim HU. Morbidity and mortality associated with traditional uvulectomy among neonates in a tertiary health facility in Kano, Nigeria. Nigerian. Journal of Perinatal and Neonatal Care. 2023 Mar 14;3(1):22-8.

15. Kefelew E, Yihun M, Dawite F, Kadi A, Tofik E, Abera A, Demise K, Abebe M. Magnitude of Uvulectomy and Associated Factors Among Caregivers Having Under Five Children in Arbaminch Town Public Health Facility, Gamo Zone, Southern Ethiopia, 2022.
16. Prual A, Gamatie Y, Djakounda M, Huguet D. Traditional uvulectomy in Niger: a public health problem? Social Science & Medicine. 1994 Oct 1;39(8):1077-82.
17. Suleiman O A. Evaluation of the perceived benefits of traditional uvulectomy among a cohort population in Ilorin, Nigeria. Faculty of Otorhinolaryngology 2007.
18. Mitke Y B. Bloody traditional procedures performed during infancy in the oropharyngeal area among HIV+ children: implication from the perspective of mother-to-child transmission of HIV. AIDS and Behavior. 2010 Dec;14:1428-36.
19. Ajibade B L, Okunlade JO, Kolade OA. Harmful cultural practices: parents perceived effects of traditional uvulectomy on the under-five-children in Jigawa State, Nigeria. Journal of Dental and Medical Sciences. 2013;9(5):8-13.
20. Farouk Z L, Slusher TM, Danzomo AA, Slusher IL. Factors influencing neonatal practice in a rural community in Kano (Northern), Nigeria. Journal of tropical pediatrics. 2019 Dec;65(6):569-75.
21. Isa A, Omotara BA, Sandabe MB, Garandawa HI. Parental reasons and perception of traditional uvulectomy in children. Sahel Medical Journal. 2011 Dec 1;14(4):210.
22. Fentaw K D, Fenta SM, Biresaw HB, Yalew MM. Factors associated with post-neonatal mortality in Ethiopia: Using the 2019 Ethiopia mini demographic and health survey. Plos one. 2022 Jul 27;17(7):e0272016.

23. Tolossa T, Fekadu G, Mengist B, Mulisa D, Fetensa G, Bekele D. Impact of antenatal care on neonatal mortality among neonates in Ethiopia: a systematic review and meta-analysis. Archives of Public Health. 2020 Dec;78(1):1-11.
24. Sawe H R, Mfinanga JA, Ringo FH, Mwafongo V, Reynolds TA, Runyon MS. Morbidity and mortality following traditional uvulectomy among children presenting to the Muhimbili National Hospital Emergency Department in Dar es Salaam, Tanzania. Emergency Medicine International. 2015 Jun 16;2015.
25. Kebede K MK, Chekole M, Zewdie A. Prevalence and Associated Factors to Uvula Cutting on Under Five Children in Amhara Region, Debre Birhan Town, 2016/2017
26. Abebe H, Beyene GA, Mulat BS. Harmful cultural practices during perinatal period and associated factors among women of childbearing age in southern Ethiopia: community based cross-sectional study. PLoS One. 2021 Jul 2;16(7):e0254095.
27. Alene G D, Edris M. Knowledge, attitudes and practices involved in harmful health behavior in Dembia district, Northwest Ethiopia. Ethiopian Journal of Health Development. 2002;16(2):199-207.
28. Adoga A A, Nimkur TL. The traditionally amputated uvula amongst Nigerians: still an ongoing practice. International Scholarly Research Notices. 2011;2011.
29. Kufa A. Harmful traditional practices (HTP). An analysis of its prevalence of and associated factors among children in Ethiopia. GRIN Verlag; 2019 Apr 8.
