# Supplementary material for: Prevalence of traditional uvulectomy and its associated factors among parents who had less than 6 months of infants in Gondar City, north-west Ethiopia: Mixed study design
Source: PLoS One. 2024 Jul 5;19(7):e0305175. doi: 10.1371/journal.pone.0305175 (PMC11226079; doi:10.1371/journal.pone.0305175)
Supplement: Attachment — Submitted filename: plos one.docx [file pone.0305175.s001.docx]

Hi,

Dear editor of Plos One

The valuable manuscript with the title " Prevalence of traditional uvulectomy and its associated factors among parents who had less than 6 months of infants in Gondar city, north-west Ethiopia: Mixed study design" was reviewed, while congratulating the authors for conducting this valuable research, some points for corrections are stated.

**Title:** The title could be shorter and stated as follows:

"Prevalence of traditional uvulectomy and its associated factors in Gondar city, north-west Ethiopia: Mixed study design"

And in the work method, parents with babies less than 6 months old should be included as entry criteria.

**Abstract**: Method: Explain about the data collection tool

Line 43: In the abstract it is not necessary to say "escriptive statistics were computed and presented using tables, figures and texts."

**Background**: Write the introduction a little more briefly

**Methods**:

Line 180-182: In the quantitative part of the study, how many questions did the data collection tool have? Except for demographic characteristics, about other components such as knowledge and attitude, if there is a question, state the items.

Line 183: Explain how to score the questions and the validity and reliability of the data collection tool

Line 196: For qualitative part of the study, the in-depth interview guide what parts did it include?

Line 201: Where was the location of the interviews?

Line 228: How many people coded the qualitative data and categorized them?

**Result**

Line 347: In a table, report the main and sub-themes from the qualitative study.

**Discussion**

It would have been better to have at least one paragraph discussing only the results of the qualitative study

**Conclusion**

In the conclusion, suggestions and solutions based on the results of the present study should be presented

**References**

You could use new references like the following in the discussion

1-Alebachew Bayih W, Minuye Birhan B, Yeshambel Alemu A. The burden of traditional neonatal uvulectomy among admissions to neonatal intensive care units, North Central Ethiopia, 2019: A triangulated crossectional study. PLoS One. 2020 Jul 9;15(7):e0234866. doi: 10.1371/journal.pone.0234866. PMID: 32645108; PMCID: PMC7347129.

2-Abdalla D, Mohammed Z, Satti E, Osman A, Alrawa SS, Abdalla S, Ibrahim D, Balla SA, Hussien Mohamed Ahmed KA. Traditional uvulectomy; practice and belief among Sudanese children in Khartoum hospitals: A multi-center cross-sectional study. Int J Pediatr Otorhinolaryngol. 2024 Jan;176:111822. doi: 10.1016/j.ijporl.2023.111822. Epub 2023 Dec 9. PMID: 38091878.
