# Supplementary material for: Prevalence of traditional uvulectomy and its associated factors among parents who had less than 6 months of infants in Gondar City, north-west Ethiopia: Mixed study design
Source: PLoS One. 2024 Jul 5;19(7):e0305175. doi: 10.1371/journal.pone.0305175 (PMC11226079; doi:10.1371/journal.pone.0305175)
Supplement: Attachment — Submitted filename: Response to reviewer.docx [file pone.0305175.s003.docx]

**PLOS/One**

From Lakachew Yismaw Bazezew

E- mail: [lakachewyismaw@gmail.com(1)](mailto:lakachewyismaw@gmail.com(1))

**Subject: submitting response to reviewers’ comments and questions**

**Title**: - Prevalence of traditional uvulectomy and its associated factors among parents who had less than 6 months of infants in Gondar city, north-west Ethiopia: Mixed study design

First of all, we would like to thank reviewers for your indispensable comments and supports to forward our manuscript. We included all constructive reviewers’ comments. We have revised all things made point by point in accordance with the comments and questions of reviewers. Please notice that we included the responses to each reviewer’s comments and questions in the following responses.

- **Responses to reviewers’ Comments and questions**

**Responses to reviewer 1**

- **Reviewer’s comments:** The title could be shorter and stated as follows: "Prevalence of traditional uvulectomy and its associated factors in Gondar city, Northwest Ethiopia: Mixed study design”
- **Authors’ response:** We thank reviewer’s ( #1) constructive comment , and Considered reviewer’s recommendation, we considered both of reviewer’s comments and the manuscripts objectives of the title, and we have used the previous title as it was and the second reviewer recommended that the title continue as it was (Prof. Tanimola Makanjuola Akande or Reviewer #2) : Title: Prevalence of traditional uvulectomy and its associated factors among parents who had less than 6 months of infants in Gondar city, north-west Ethiopia: Mixed study Design (see page 1, line 1-3)
- **Reviewer’s comments and questions:** in the work method, parents with babies less than 6 months old should be included as entry criteria.
- **Authors’ response:** we appreciate reviewer’s comment and we accepted the comment we already considered parents with babies less than 6 months old have been included as entry criteria in the work method.(see page 7,Line:162-163
- **Reviewer’s comment:** Abstract: Method: Explain about the data collection tool
- **Authors’ response:** we thank reviewer for your comment, and we agreed with reviewer’s comment, and we have explained the data collection tool at the method section of abstract.(see page 2,Line:40-41)
- **Reviewer’s comments:** In the abstract it is not necessary to say "escriptive statistics were computed and presented using tables, figures and texts."
- **Authors’ response:** We thank you very much, and appreciated reviewer’s for their valuable focus to correct, and forward our manuscript and we corrected capitalization, spelling error in the abstract the word “escriptive” to descriptive. (see page 2,Line:39 and 43)
- **Reviewer’s comments** :Background: Write the introduction a little more briefly
- **Authors’ response:** We thank you, agreed with reviewer comment, and we wrote the introduction a little more briefly.( see page 4,Line:79-83)
- **Reviewer’s suggestion and questions**: In the quantitative part of the study, how many questions did the data collection tool have? Except for demographic characteristics, about other components such as knowledge and attitude, if there is a question, state the items.
- **Authors’ response:** We accepted reviewer comment and addressed question as follows:, there are main questions which are **infrastructure related question like**: 1.no nearby health institution, 2.lack of better medical care, 3.presence of traditional practitioner, **obstetric related questions like**: 1.number of ANC visits, 2.Antenatal counseling of traditional uvuloctomy, 3.ever had bad obstetrics history, 4.Place of delivery, 5.Number of PNC visits, 6.Postnatal counseling about traditional uvulectomy, 7.Parity of mother, 8.ever had Breastfeeding ,9. Hospitable health professional. **Awareness related questions such as**: 1.Information about traditional uvuloctomy, 2.Source of information about uvulectomy, 3.Information about uvula, 4.source of information about uvula 5.information about benefit of uvulectomy, **practice related question such as**: 1.ever had Traditional uvulectomy, 2. Age of infant during uvulectomy practice, 3. Cost of uvulectomy, 4.Complication of uvulectomy, 5.Types of complication, and **Attitude related questions, such as:** 1. Do you think that Uvulectomy causes disease or illness? 2. Do you think that traditional Uvulectomy is harmful? Do you perform traditional uvulectomy in the future? 4. Do you encourage other people to cut their baby’s uvula? 5. Do you think that uvula cutting should be eradicated? 6. Reason to seek traditional uvulectomy.
- **Reviewer’s questions**: Explain how to score the questions and the validity and reliability of the data collection tool?
- **Authors’ response:** To address reviewers recommendation and questions the way of scoring the questions are as following: Awareness: awareness related issues of the respondent was assessed by five yes/no awareness related questions which was measured separately from the report of caregivers and coded 1 if the caregivers reported yes for the given awareness related information; otherwise, it was coded 0 for an unwearied information. Attitude (Good, Poor): The attitude of the participants toward Uvulectomy was assessed using 5 questions with yes, and no responses. At first, questions 3 and 4 of attitude questions were recoded (1 to 0 and 2 to 1) to bring similar responses/meanings/, Then those 5 questions were computed for making one category (attitude) and attitude was grouped into two (good and poor) using the mean score as a cutoff point. Those with less than or equal to the mean score of 5 yes /no attitude related questions were coded as having a good attitude and the rest as having a poor attitude. Validity and reliability of data collection tool insured by choose appropriate tools, Designed and tested instruments, Implemented and monitored instruments, analyzed and reported instruments. We used revalidated tools taken from different literatures. Selected most suitable research question, designed, and selected representative population to ensured validity and reliability of data collection tools. We considered the type, level, and scope of data that we need, as well as the feasibility, cost, and ethics of using different tools. We have used pretest through closed-ended questions that can be easily administered and analyzed. On the other hand when we want to explore the experiences and meanings of informants, we used an interview with open-ended questions that can elicit rich and detailed responses for qualitative parts of study. We Designed and tested our instruments carefully before using them in our actual research to ensure the validity and reliability of our data collection tools. We have used clear and simple language, avoided bias and ambiguity, provided instructions and we have tested our tools on a sample of our target population or experts in our field to check for any errors, misunderstandings, or difficulties that might affect the validity and reliability of our data. We have used techniques such as pretest and expert review to evaluate and improve our instruments. We Implemented and monitored our instruments in a consistent and standardized way in our actual research to ensure the validity and reliability of our data collection tools. such as selecting a representative and random sample, obtaining informed consent, providing clear and accurate instructions, maintaining rapport and confidentiality, and avoided interference or influence. We also monitored our instruments throughout the data collection process, such as recording any problems, deviations, or feedback that might affect the validity and reliability of our data. We have used methods such as quality control, quality assurance, to check and ensure the quality of our data collection tool. We Analyzed and reported our tool in a transparent and rigorous way in our research report to ensure the validity and reliability of our data collection instruments. We have used appropriate and valid statistical methods to analyze our data, such as descriptive statistics, and factor analysis. We have acknowledged any limitations, challenges that might affect the validity and reliability of our data collection tools, and suggested ways to overcome or address them in future research.
- **Reviewer’s questions**: For qualitative part of the study, the in-depth interview guide what parts did it include? What types of interview is this? How is it different from KIIs?
- **Authors’ response:** we thank and appreciated for support to forward our manuscript, to address reviewer question, it has two parts, the first part is engaging ,and the second is probing Semi-structured guiding questioners which was applied for key and individual in-depth informants accordingly.to clarify their deference ,individual depth interview focused on the gathering information from individuals own self experience, reasons and perceptions to their real practice of the study participants ,and KIIs is interview that focused on the experience, reason ,and perceptions of the practice on behalf of the study participants, and have the knowledge and practice on the phenomenon .
- **Reviewer’s questions**: Where was the location of the interviews?
- **Authors’ response:** we appreciated reviewer and to address reviewer’s question, and the interview was conducted at participants home that was free from any disturbance), audio recording sound, and time frames were maintained appropriately.
- **Reviewer’s comments and questions:** How many people coded the qualitative data and categorized them?
- **Authors’ response:** Ten individual depth interviews from mothers who had less than 6 months old children and five key informants were included for qualitative part of the study, Three health extension workers and two traditional healers were participated as key informants. Name of mothers, health extension workers and traditional healers was coded as I-01 to I-10, K-01 to K-03 and K-04 to K-05 respectively.(see page 8,Line:175 to 180)
- **Reviewer’s comments and questions:** In a table, report the main and sub-themes from the qualitative study.
- **Author’s response:**
  Thank you for your constructive comment, and we accepted the comment and reported the main and sub-themes in a table at main document.(see page 21,Line:434 to 436 )
- **Reviewer’s comments and questions**: It would have been better to have at least one paragraph discussing only the results of the qualitative study.
- **Authors’ response:** We would like to thank reviewer for your constructive comments, and we had discussed only the result of qualitative study in a paragraph at discussion part. (see page 24-25,Line: 505 -515
- **Reviewer’s comments:** In the conclusion, suggestions and solutions based on the results of the present study should be presented.
- **Authors’ response:** We accepted the reviewer constructive comments and we presented suggestions and solutions based on the results of the present study at main documents.(see page25 ,line 525-528)
- **Reviewer’s comment:** It would have been better to have at least one paragraph discussing only the results of the qualitative study
- **Authors’ response:** We accepted and appreciated the reviewer comment and the reference is updated at discussion part of manuscript which was available in the main documents.( see page22 ,line 448 to 450 )
- **Reviewer’s comment:** The ethics approval code should be written
- **Authors’ response:** We accepted reviewer comments, and appreciated your support, we have written ethics approval code at main documents at ethics parts of it as Ref.N.o:037/2015) october03/2022.(see page10 ,Line:241-242)

Response to reviewer 2

- **Reviewer’s comments:** **Title: Prevalence of traditional uvulectomy and its associated factors among parents who had less than 6 months of infants in Gondar city, north-west Ethiopia: Mixed study design. The title is in line with study objectives and method. Abstract: Well-structured**
- **Minor typographical error.**
- **Authors’ response:** We thank you very much for your constructive support and comments to forward our manuscript, and we took your accepted point of view as it was and corrected topographical errors.(see page 2 ,Line:39,40 ,41and 43)
- **Reviewer’s comments:** Background: Detailed information on statement of problem, rational for the study and study objectives are clearly. Some grammatical errors highlighted for correction.
- **Authors’ response:** We thank you, took your accepted point of view as it was, accepted the comment and corrected grammatical errors of background highlighted for correction.( see page 2,Line:39 and 43)
- **Reviewer’s comments and questions:** Methods: Fairly well described. Selection of participants by systematic sampling - This is not clear.
- **Authors’ response:** Thank you very much for your support, took your accepted parts of manuscript as it was, to clarify the sampling techniques which we used, the Selection of participants were: by using lottery method nine kebeles were selected from 36 kebeles of Gondar city and based on the total number of caregivers having children less than 6 months in each kebeles the study sample proportional allocation was made. Finally, the participants were selected using systematic random sampling techniques after leveled of each household which has mother who had less than 6 months old child in each selected kebeles.
- **Reviewer’s questions and comments:** Was there a sampling frame for the communities? This is a community-based study. It is unlikely to select respondents in a city without the use of multi-stage random sampling.
- **Authors’ response:** we thank the reviewer, to address the question, it is difficult to say as sampling frame for the community because getting list of population is mandatory due to that reason we have used systematic sampling techniques which does not need to have list of population.(see page7,line170-171)
- **Reviewer’s questions and comments**: Provide the total number of KIIs and how they were distributed among health extension workers and traditional healers.
- **Authors’ response:** Thank you for your recommendation, we accepted it, the total number of key informants was 5 who were categorized as health extension workers and traditional healers. They were selected purposively, and who were not involved in the quantitative study.
- **Reviewer’s question:** was interview conducted among the nursing mothers?
- **Authors’ response:** we thank the reviewer, to answer the question; yes the interview was conducted among the nursing mothers.
- **Reviewer’s questions and comments:** Does this mean only one research assistant was used for qualitative study? Qualitative study requires at least a pair of research assistants for each session.
- **Authors’ response:** we appreciated reviewer for support, to address the question you may consider it as limitation for not used pair of or more research assistant but it was due to budget or feasibility issues, even if it does not mean only one research assistant was used for qualitative study, We used supervisor who was considered as research assistants for each session in addition to the assistant’s roles, the supervisor was participated as overall activity controller when the principal investigator interviewed the participants.(see page9,line 201-206)
- **Reviewer’s question:** Fairly well written in details with relevant tables and figures. Which table provided this data? No table clearly showed the prevalence of traditional uvulectomy
- **Authors’ response:** We appreciated the comment, we took your accepted point of view and we put the prevalence of traditional uvulectomy on the table to clearly show this data.(see page 16 Line:351-353)
- **Reviewer’s question:** The highlighted statement is not clear.
- **Authors’ response:** we appreciated the comment and to address it, the statement want to say mothers desired to continue to seek traditional healer due to their infants not cured by medical care even if they accessed the medical care at health institution.( see page 20,Line:417 to 418)
- **Reviewer’s comment:** The study findings are well discussed and compared with other studies. Prevalence of uvulectomy not clearly shown on any of the tables
- **Authors’ response:** We appreciated the comment, took your accepted part of manuscript as it was and we put the prevalence of traditional uvulectomy on the table to clearly show this data.(see page 16 Line:351-353)
- **Reviewer’s comment:** Check the spelling, grammatical and syntax errors highlighted for correction.
- **Authors’ response:** we thank you, and accepted reviewer comments have corrected the spelling, grammatical and syntax errors highlighted of discussion.(see page 21-23 Line:456,458,461,462,and 469)
- **Reviewer’s comment:** Clearly written but without any clear recommendation based on the findings in this study.
- **Authors’ response:** We accepted reviewer comment; took the accepted part of manuscripts as it was, put recommendation at main document of conclusion based on the findings of this study.(see page 25 Line:526 to 528 )
